# Clinical application of noninvasive prenatal screening for sex chromosome aneuploidies in 50,301 pregnancies: initial experience in a Chinese hospital

**DOI:** 10.1038/s41598-019-44018-4

**Published:** 2019-05-23

**Authors:** Cechuan Deng, Qian Zhu, Sha Liu, Jianlong Liu, Ting Bai, Xiaosha Jing, Tianyu Xia, Yunyun Liu, Jing Cheng, Zhunduo Li, Xiang Wei, Lingling Xing, Yuan Luo, Hongqian Liu

**Affiliations:** 1 Department of Obstetrics and Gynecology, West China Second University Hospital, Sichuan University,, Chengdu, 610041 China; 2 Key Laboratory of Birth Defects and Related Diseases of Women and Children, Ministry of Education, Sichuan University, Chengdu, 610041 China

**Keywords:** Chromosome abnormality, Sequencing

## Abstract

To evaluate the clinical performance of noninvasive prenatal screening (NIPS) for fetal sex chromosome aneuploidies (SCAs), pregnant women were recruited in this retrospective observational study. The NIPS test was undertaken using high-throughput gene sequencing. In total,50,301 pregnant women were analysed for demographic characteristics and medical history. Of them, 308 women (0.61%) had high risk for fetal SCAs, including 138 for 45,X, 111 for 47,XXY, 42 for 47,XXX, and 17 for 47,XYY. After the pre-test counselling, 182 participants chose to undergo invasive prenatal diagnosis, confirming 59 positive cases. The combined positive predictive value of NIPS was 32.42% (59/182), 18.39% (16/87), 44.4% (12/27), 39.29% (22/56), and 75% (9/12) for detecting SCAs, 45,X, 47,XXX, 47,XXY, and 47,XYY, respectively. NIPS can be a useful method to detect the fetal SCAs using high-throughput gene sequencing, though accuracy can still be improved, especially for 45,X. Although the value of NIPS compare favorably with those seen in traditional screening approaches for SCAs, it is important to highlight the limitations of NIPS while educating clinicians and patients.

## Introduction

Birth defects or congenital anomalies refer to anomalies in the anatomy and function of an embryo or fetus during its development due to genetic factors, including chromosomal and genetic anomalies. Birth defects are the main cause of death, illness, disability and poor quality of life of children. Chromosome abnormality is an important cause of birth defects. Errors in the execution of maternal or paternal meiosis can lead to chromosome aneuploidy. Among the most common aneuploidies compatible with live birth are those involving the X and Y chromosomes. X or Y chromosome abnormality, also known as sex chromosome abnormality (SCA), can cause male or female sexual organ dysplasia. Patients with SCA may have structural or functional abnormalities in other organs, or may demonstrate other clinical manifestations, such as certain intellectual disability or mental neurological disorders^1^. Triple X syndrome (47,XXX), Turner syndrome (45,X), Klinefelter syndrome (47,XXY), and XYY syndrome (47,XYY) are common manifestations of abnormal sex chromosome, with phenotypes, including low reproductive ability, infertility, and language development retardation. The combined frequency of these disorders ranges from 1/500 to 1/850 for male and female fetuses, respectively^2,3^. This relatively high incidence makes prenatal screening and diagnosis of SCA’s an attractive option for pregnant women.

Invasive prenatal diagnosis by amniocentesis or by cordcentesis is the gold standard for the diagnosis of SCA. But because of the higher incidence rates of fetal loss, infections^4^, and the mental stress caused to the pregnant women and their families, more doctors and pregnant women tend to prefer initial noninvasive screening than the invasive prenatal diagnosis. Down syndrome (DS) screening is the routine prenatal biochemical screening, which can calculate the risk of fetus trisomy 21 (T21), trisomy 18 (T18) and open neural tube defect by detecting the levels of free β-subunit of human chorionic gonadotropin in maternal serum (fβhCG), alpha fetoprotein (AFP) and unconjugated estriol (uE3) combined with other information about the pregnant women^5,6^. The detection rate is 75% for trisomy 21 and the false positive rate is 5% with the routine biochemical prenatal screening program^7^. However, this method is not suitable to screen for SCAs.

Lo *et al*.^8^ detected the presence of circulating cell free fetal DNA (cffDNA) in the blood samples of pregnant women using sensitive Y-PCR. This discovery opened a new chapter in non-invasive prenatal screening (NIPS), also known as noninvasive prenatal testing (NIPT). The birth of the next-generation sequencing technology (NGS) has enabled the clinical application of cffDNA. In recent years, noninvasive prenatal screening based on massively parallel genomic sequencing (MPS) technology has been widely applied for the clinical detection of trisomy 21, trisomy 18, and trisomy 13^9^. A meta-analysis conducted in 2015 showed that in single pregnancy, the detection rate of NIPS was 99.2% for trisomy 21, 96.3% for trisomy 18, and 91.0% for trisomy 13; the false positive rate was 0.09% for trisomy 21, 0.13% for trisomy 18, and 0.13% for trisomy 13^10^. More reports have come up with similar outcomes^11,12^. However, there are few studies on the noninvasive prenatal testing of sex chromosome abnormalities. In this study, MPS was used to detect cffDNA in the peripheral blood of pregnant women. Amniotic fluid or cord blood karyotype analysis was performed in pregnant women with the NIPS result of sex chromosomal abnormalities. This study aims to evaluate the clinical performance of NIPS for detecting fetal SCAs using MPS.

## Results

**Figure 1:**
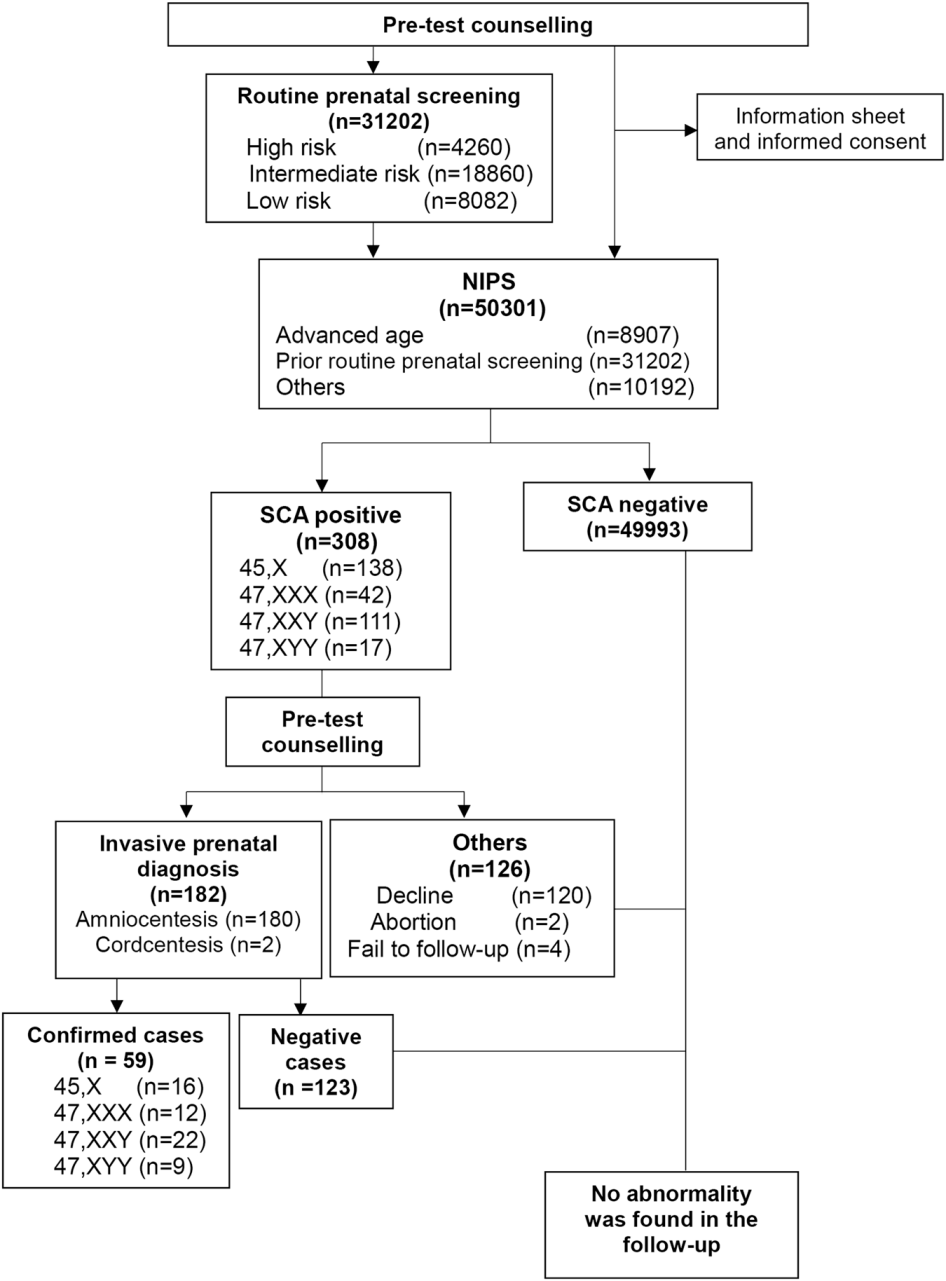
Clinical consultation process of the study evaluating the use of NIPS for detecting fetal SCAs.

All the 50,301 pregnant women were of Chinese ethnicity, presenting for NIPS at WCSUH between May 2015 and September 2017, and consented to participate in the study. Of these, 19,099 pregnant women chose to perform NIPS without prior routine biochemical prenatal screening, and 31,202 chose to undergo routine prenatal biochemical screening before NIPS. The entire process of study is showed in Fig. 1. Patient characteristics are summarised in Table 1. A total of 8,907 of 50,301(17.71%) participants were 35 years or older. A total of 4,260 of 50,301 (8.47%) participants were at high risk for DS, and 18,860 of 50,301 (37.49%) participants were at intermediate risk for DS based on routine prenatal biochemical screening.

**Table 1:** Patient characteristics of 50,301 singleton pregnancies undergoing NIPS for chromosomal aneuploidy.

Characteristic	n (%)
**Maternal age**	
<35 years	41394 (82.29)
≥35 years	8907 (17.71)
**Gestational age**	
12–22^+6^ weeks	43931 (87.34)
>22^+6^ weeks	6370 (12.66)
**Ethnicity**	
Chinese	50301 (100.00)
Singleton pregnancy	50301 (100.00)
**Result of prenatal biochemical screening**	
High risk	4260 (8.47)
Intermediate risk	18860 (37.49)
Low risk	8082 (16.07)
Not performed	19099 (37.97)

### Clinical results of NIPS

There were 308 cases classified as SCA-positive by the NIPS, including 138 positive NIPS results for monosomy X, 42 for XXX, 111 for XXY, and 17 for XYY. The mean ± SD maternal age of the 308 patients with SCA-positive NIPS results was 29 ± 5 years (51 cases were 35 years or older) and the mean gestational age at testing was 20 ± 3 weeks.

After the pre-test clinical counselling, all 308 (100%) participants agreed to know the risk of having SCAs in their fetus (Table 2). Total 182 of 308 (59.09%) informed women underwent invasive prenatal diagnosis, including 180 cases of amniocentesis and 2 cases of cordocentesis. However, 122 (41.56%) participants did not undergo either invasive procedure because they declined further studies (n = 120), or the fetus was aborted (n = 2) (Table 2).

**Table 2:** Clinical results of 50,301 singleton pregnancies using NIPS for detecting foetal SCAs.

SCA type	NIPS SCA detected	Woman followed up	Invasive prenatal diagnosis	
			Performed	Declined
Total	308	304*	182 (60)	122 (40)
X0	138	135	87 (64)	48 (36)
XXX	42	42	27 (64)	15 (36)
XXY	111	110	56 (51)	54 (49)
XYY	17	17	12 (71)	5 (29)

Data are given as *n* or *n* (%). *In three X0 samples and one XXY sample we failed to obtain karyotyping results from amniocentesis because of cell culture failure.

### Comparison between NIPS and prenatal diagnosis for detecting SCAs

A total of 182 of the 308 pregnant women with a SCA-positive NIPS result chose to undergo invasive prenatal diagnosis, and 59 cases were confirmed as SCA-positive by karyotyping (16 cases of monosomy X, 12 of XXX, 22 of XXY, and 9 of XYY, while the remaining 123 cases had normal karyotypes. Three of these cases had normal fetal karyotype, but had 47,XXX as maternal karyotype. The combined positive predictive value (PPV) of NIPS was 32.42% (59/182) for detecting fetal SCAs (Table 3). The PPV for individual SCA was as follows: XYY syndrome (47,XYY; 75% [9/12]), Triple X syndrome (47,XXX; 44.4% [12/27]), Klinefelter syndrome (47,XXY;39.9% [22/56]), and Turner syndrome (45,X; 18.39% [16/87]). All cases of negative NIPS were followed up and no false-negative results were identified. The sensitivity and specificity of NIPS for detecting fetal SCAs was 100% [59/59] and 99.5% [49993/50242], respectively.

**Table 3:** Comparison between NIPS and karyotyping for detecting fetal SCAs.

SCA type	NIPS SCA detected	Confirmation by karyotype			PPV	FPR
		Yes	No	N/A		
Total	308 (0.61)	59 (0.12)	123 (0.24)	4 (0)	32.42	67.58
X0	138 (0.27)	16 (0.03)	71 (0.14)	3 (0)	18.39	81.61
XXX	42 (0.08)	12 (0.02)	15 (0.03)	0 (0)	44.40	55.60
XXY	111 (0.22)	22 (0.04)	34 (0.07)	1 (0)	39.29	60.71
XYY	17 (0.03)	9 (0.02)	3 (0.01)	0 (0)	75.00	25.00

Data are given as *n* (%) or %. N/A, not available. PPV, positive predictive value of a prenatally confirmed SCA after an invasive test for a positive NIPS result. FPR, false-positive rate.

### Comparison between NIPS and prenatal diagnosis based on stratification of demographic characteristics

The comparison between NIPS and karyotyping for fetal SCAs in participants based on stratification of demographic characteristics, including the risk of routine prenatal biochemical screening, age of pregnant women, gestational age, and maternal BMI is shown in Table 4. It was confirmed that 28 (0.65%) of the 4260 participants with high risk as per routine prenatal biochemical screening had positive results of NIPS. After verification of karyotype analysis, five participants were confirmed as true positive, generating a positive predictive value of 38.46%. A total of 108 (0.57%) of the 18,860 participants with intermediate risk as per routine prenatal biochemical screening had positive results of NIPS, 22 of whom were confirmed true positive, generating a positive predictive value of 34.92%. Similarly, 55 (0.68%) of the 8082 participants with low risk as per routine prenatal biochemical screening had positive results of NIPS, 8 of whom were confirmed true positive, generating a positive predictive value of 25.81%. The PPV of low-risk group was lower than that of high-risk and intermediate-risk groups.

**Table 4:** Comparison between NIPS and karyotyping for detecting foetal SCAs based on stratification of demographic characteristics.

Characteristic	n	NIPS positive	Without karyotype validated	Karyotype validated^a^		PPV (%)
				TP	FP	
**Prenatal biochemical screening**						
High risk	4260	28	15	5	8	38.46
Intermediate risk	18860	108	43	22	41	34.92
Low risk	8082	55	22	8	23	25.81
Not performed	19099	117	42	24	51	32.00
**Maternal age**						
<35 years	41394	250	98	49	101	32.67
≥35 years	8907	58	24	10	22	31.25
**Gestational age**						
12–22 + 6 weeks	43931	251	96	54	97	35.76
≥22 + 6 weeks	6370	57	26	5	26	16.13
**BMI**						
<18.5	1095	17	5	5	6	45.45
18.5–27.9	48550	269	107	52	108	32.50
≥28	656	22	10	2	9	18.18

^a^Tests included amniocentesis and cordocentesis karyotyping. Data are given as *n*. BMI (kg/m^2^)^29^: Body Mass Index = weight(kg)/height(m)^2^. TP, True positive. FP, False positive. PPV, positive predictive value.

Moreover, 10 of the 58 participants with advanced age and positive results as per NIPS were confirmed true positive by invasive prenatal diagnosis, generating a positive predictive value of 31.25%.

Similarly, 54 of the 251 participants with gestational age <22 + 6 weeks and positive results as per NIPS were confirmed true by invasive prenatal diagnosis, generating a positive predictive value of 35.76%. In addition, 5 of the 57 participants with gestational age ≥22 + 6 weeks and positive results as per NIPS were confirmed true by invasive prenatal diagnosis, generating a positive predictive value of 16.13%, which is much lower than the PPV of gestational age <22 + 6 weeks.

Similarly, 5 of the 17 participants with BMI < 18.5 and positive results as per NIPS were confirmed by invasive prenatal diagnosis, giving a positive predictive value of 45.45%. Total 52 of the 269 participants with BMI ranging from 18.5 to 27.9 and positive results as per NIPS were confirmed by invasive prenatal diagnosis, giving a positive predictive value of 32.5%. For the women with BMI ≥ 28, the PPV was 18.18%, which is much lower than the PPV of BMI < 18.5 and BMI from 18.5 to 27.9.

### Comparison of stratified demographic characteristics with the results of karyotyping validation

We carried out statistical analysis of the SCA-positive cases by NIPS and karyotyping validation in participants based on stratification of demographic characteristics, including the risk of routine prenatal biochemical screening, age of pregnant women, gestational age, and maternal BMI. There was no significant difference associated with routine prenatal biochemical screening risk (*P* = 0.796), maternal age (*P* = 0.876), and BMI (*P* = 0.392) (Table 5). However, a significant difference was observed between the gestational age ≥22 + 6 weeks and <22 + 6 weeks (*P* = 0.033).

**Table 5:** Comparison between different stratified demographic characteristics with the results of karyotyping validation.

Characteristic	NIPS positive	Karyotype validated^a^		*P*
		TP	FP	
**Prenatal biochemical screening**				
High risk	28	5	8	
Intermediate risk	108	22	41	
Low risk	55	8	23	
Not performed	117	24	51	0.796
**Maternal age**				
<35 years	250	49	101	
≥35 years	58	10	22	0.876
**Gestational age**				
12–22 + 6 weeks	251	54	97	
≥22 + 6 weeks	57	5	26	0.033
**BMI**				
<18.5	17	5	6	
18.5–27.9	269	52	108	
≥28	22	2	9	0.392

^a^Tests included amniocentesis and cordocentesis karyotyping. Data are given as *n*. BMI (kg/m^2^)^29^: Body Mass Index = weight(kg)/height(m)^2^. TP, True positive. FP, False positive^5^.

## Discussion

NIPS is a noninvasive prenatal test based on fetal free DNA in the serum of pregnant women and using second generation sequencing technology. NIPS is noninvasive compared to the traditional prenatal diagnosis, so can avoid potential fetal loss and infection due to puncture^4,13^. As compared with routine prenatal biochemical screening, NIPS has higher sensitivity and specificity for the detection of common chromosomal aneuploidies, such as trisomy 21, trisomy 18, and trisomy 13. A previous research has shown that for trisomy 21, NIPS had 97.95–100.00% detection specificity, 98.58–100.00% sensitivity, and 0.3% false positive rate^14,15^. The detection rates of NIPS was 100.00% for trisomy 18, and 91.70% for trisomy 13, with false positive rates 0.28% and 0.97%, respectively^16^. More reports have come up with similar outcomes^11,12^.

Some studies have reported that NIPS can be used to detect SCAs, apart from detecting trisomy 21, trisomy 18, and trisomy 13. However, the detection accuracy of sex chromosome aneuploidy using NIPS varies among different research groups. For instance, in a meta-analysis, the detection rates of NIPS for monosomy X and for other SCAs except monosomy X were 93.0% and 90.3%, respectively^10^. Nevertheless, detection rates varied across different studies in this analysis. For example, the lowest and the highest detection rates for monosomy X were 66.7% and 100%, respectively^10^. A previous research indicated that NIPS using massively parallel genomic sequencing has a high sensitivity (92.6%) and a low false positive rate (<1%) for screening foetal SCAs^17^. One research by a target assay reported that the PPV was 48.4% and the negative predictive value was 100% for SCAs^18^. Another similar study showed that the PPV for SCAs was 54.17%^19^. Y. Xue *et al*. found that the over specificity and PPV were 99.90% and 57.1% for Proton, and 99.78% and 36.9% for illumina, respectively, for fetal SCAs^20^.

In this study, NGS was used to evaluate the clinical performance of noninvasive prenatal screening for fetal sex chromosome aneuploidies. NIPS results indicated 308 cases of suspected SCAs, and invasive prenatal diagnosis confirmed 59 of those cases as SCAs. The combined PPV of NIPS in this study was 32.42%, and the PPV for individual SCAs was as follows:18.39% for detecting 45,X, 44.4% for 47,XXX, 39.29% for 47,XXY, and 75% for 47,XYY. In the study, 4 participants failed to follow up, and 122 of the 304 viable pregnancies (40%) with a positive SCA result by NIPS declined prenatal diagnosis. If the results of the 126 pregnant women had been confirmed as true positive, the highest combined positive predictive value of NIPS would have been 60.06% (185/308); and if the results of those participants had been demonstrated as false positive, the lowest combined positive predictive value of NIPS would have been 19.16% (59/308).

In our population, NIPS appeared to more accurately predict triple X and XYY syndrome, but performed poorly as a predictor of fetal monosomy X. There are several possible reasons: (i) X and Y chromosome have 58 homologous genes, 29 genes of which are located at both ends of X and Y chromosomes-the pseudoautosomal region. The pseudoautosomal region consists of two short segments at both ends of sex chromosomes. An error in sequencing these locations on X and Y chromosomes might easily happen because of the short sequencing length of 36 bases of non-invasive prenatal screening; (ii) Contributing maternal factors include X chromosome mosaicism in pregnant women^21^_._ The PPV of NIPS for an affected fetus with 45,X was 18.39% in our study, lower than that reported by others^10,19,22,23^_._ Yao *et al*.^19^ and Bianchi *et al*.^22^ found a PPV of 20% for NIPS predicting monosomy X. However clinical follow-up was available in only one-third of pregnancies tested positive by NIPS in their studies. The PPV of any test will be lower in populations with low prevalence of the disorder. Comparing the invasive diagnostic testing without NIPS in all the cases, except a confirmed 45,X case, prompted by the detection of a thickened nuchal translucency (NT), our NIPS-tested population had a lower background prevalence. If NT is more than 3 mm, diagnostic testing, rather than NIPS screening, is usually recommended. Thus, a low prevalence of monosomy X would be a trait of any population in which NT was performed prior to NIPS.

There are more intrinsic biological reasons for inconsistent results between NIPS and invasive diagnosis testing that can be of either fetal or maternal origin. Contributing fetal factors include an absent or insufficient fetal DNA fraction, the karyotype inconformity of the fetus, mosaicism, confined placental mosaicism, and the presence of a vanishing twin^24^. CffDNA is mainly derived from the trophoblast cells in the apoptotic layer of placental villi, which are not always representative of the fetal situation due to mosaicism. The level of cytotrophoblastic chimerism and the sensitivity of the detection method for low proportion chimeras may influence the results of NIPS. The possible existence of chimeras is one of the limitations of NIPS. For instance, Hall AL *et al*. reported a case that confirmed positive cffDNA testing for trisomy 13, demonstrated to be confined placental mosaicism^25^. Contributing maternal factors include abnormal chromosome chimerism in pregnant women^21^, maternal SCAs, copynumber variation, and maternal neoplastic conditions can affect the accuracy of NIPS. In our study, there were 2 cases with the outcome of fetal triple X by NIPS but showed normal karyotype by prenatal diagnosis. Subsequently, the maternal peripheral blood chromosomes were proven to have triple X. Additional factors, such as maternal-fetal admixture, and genomic resolution are also challenges to the technology.

It was confirmed that 59 pregnant women had SCAs, and the screening and diagnosis coincidence rate was 32.42%. Of the 59 cases of SCAs, 1 case was 46,X, del(X),(q24) and 1 case was 46,XY,(arr [hg19] Yq 11.222(20467818–21028944)x3), indicating that NIPS has a certain clinical value for the detection of microdeletions and microduplications.

Though our study has many strengths, such as larger sample size consists of 50,301 cases with 308 cases of SCAs, there are several study limitations. First, we could not obtain the information about maternal or placental mosaicism contributing to the false positive rate. Second, there was a lack of postnatal data for those who elected to continue pregnancies but delivered at hospitals outside of our system. Though counselling clinicians of these patients are typically contacted in prenatal and postnatal phases to coordinate or obtain diagnostic testing results, many are still lost.

ACOG(American College of Obstetricians and Gynecologists/Society for Maternal-Fetal Medicine) practice bulletin 163^26^ states that all women should be offered the option of aneuploidy screening or diagnostic testing for fetal genetic disorders, regardless of maternal age. ACMG (American College of Medical Genetics and Genomics) recommends informing all pregnant women, as part of pre-test counselling for NIPS, of the availability of the expanded use of screening for SCAs^27^. The data presented here remind us that NIPS can be used to screen for SCAs, however, its performance is currently lower than that of traditional diagnostic tests. We suggest that all pregnant women should be informed that NIPS can be extended to SCA screening. As part of pre-test counselling and screening, patients should be informed that NIPS has a higher false positive rate for detecting SCAs and explain the reasons. These patients should be also informed about the option of traditional diagnostic testing along with risks and benefits of each option. Despite the controversy surrounding cffDNA screening, both sides agree that better methods for prenatal pre- and post-test counselling are needed, so that patients can better understand the choices they are making.

When the NIPS test results show a high risk of aneuploidy in the sex chromosome, the patient should be recommended to a professional geneticist. Therefore, it is important to inform the clinicians through data, such as that presented here. Diagnostic tests should be provided when NIPS screening results are positive for sex chromosome aneuploidy. Although prenatal diagnosis is more reliable for diagnosing autosomal aneuploidies, differences in phenotype in SCA patients may be subtle or inexistent. Routine physical examination may give the wrong signal, leading to patients refusing prenatal diagnoses or clinicians not providing such tests. If the prenatal diagnosis is rejected by the patient, obstetrical clinicians and pediatricians need to get deeper education on the importance of SCAs in postnatal testing.

PPV reveals that a positive test result might indicate an abnormal fetus. If the PPV is low, the value of the screening is limited in that regard. While the clinical effectiveness of any test, especially, in in the case of SCAs, is still important in case of high negative predictive values even if PPV is low, and when the test returns negative results, it can provide assurance to clinicians and patients. The data in this study may support the need to improve the cognition of the prospects and limitations of NIPS for SCAs screening.

## Subjects and Methods

### Subjects

The retrospective study was conducted from May 2015 to September 2017 in pregnant women undergoing prenatal screening and diagnosis at The West China Second University Hospital of Sichuan University (WCSUH), Chengdu, Sichuan Province, China.

The study enrolled pregnant women aged 18–50 years with a gestational age of 13–27 weeks. The last menstrual cycle and the first ultrasound were used to calculate the gestational age. Total, 50,301 singleton pregnant women were recruited in this study. All participants received pre-test clinical counselling, and were explained the contents, principles, and the advantages and limitations of the test by the clinician^28^.

The study has been approved by the Institutional Ethics Committee of Sichuan University and all participants signed written informed consent prior to the test. The research was conducted in accordance with the relevant guidelines and clinical norms.

### Routine prenatal biochemical screening

Total 5 mL elbow venous blood was collected into a BD Vacutainer sample tube (Becton, Dickinson& Co., Lakes, NJ, USA). The sample tubes were kept standing at room temperature (18–25 °C) for 30 min, centrifuged using a refrigerated centrifuge at 1800 g for 10 min, and the collected serum was stored at −20 °C as soon as possible. The fβhCG, AFP, and uE3 levels were detected using 1235 automatic time-resolved fluorescence immunoanalyser using appropriate reagents (PerkinElmer, Gaithersburg, MD, USA).

DS serological screening risk was calculated by Lifecycle4.0 (Finland Wallac Oy company) combined with the MOM (multiples of median) of fβhCG, AFP, and uE3, pregnant woman’s age, gestational age, weight, abnormal gestation, and birth. A value of ≥1 in 270 meant high risk for trisomy 21 and a value of ≥1 in 350 meant high risk for trisomy 18; a value from 1 in 271 to 1 in 1000 meant intermediate risk for trisomy 21, and a value from 1 in 351 to 1 in 1000 meant intermediate risk for trisomy 18. A risk value of <1 in 1000 meant low risk for trisomy 21 or trisomy 18. Advanced age referred to those aged 35 years or older on the expected date of confinement.

### Noninvasive prenatal screening

For all participants, 10 mL of maternal peripheral blood was collected in a cell-free™ BCT tube (Streck, Omaha, NE), according to the standard procedure. The collected blood samples were kept standing at room temperature for 30 min, before being stored at 4 °C. The specimens were centrifuged for 10 min using Eppendorf 5810R (Eppendorf, Hamburg, Germany) at 1600 g at 4 °C. Then the collected plasma was centrifuged for 10 min using Eppendorf 5430R (Eppendorf, Hamburg, Germany) at 16000 g at 4 °C and was stored at −70 °C. The blood plasma was used for DNA extraction using DNA extraction test kit (Hangzhou Berry gene diagnostic technology Co., Ltd., Hangzhou, China), and the free DNA concentration was detected using Qubit 3.0 and ExKubit dsDNA HS test kit (ExCell Biotech Co., Ltd., China). The cut-off limit for the extracted cffDNA concentration was <0.7 ng/mL, and another tube of blood specimen was required. The eligible cffDNA samples were subjected to library construction and a special index was added using non-invasive prenatal test library prep kit (Reversile Terminator Sequencing) (Hangzhou Berry gene diagnostic technology Co., Ltd, Hangzhou, China). The quality was tested using a KAPA SYBERFAST qPCR kit (KAPA Biosystems, Wilmington, MA, USA). All the libraries were pooled together and sequenced using a NextSeq CN500 massively parallel sequencing kit (High-throughput sequencing kit) and NextSeq CN500 high-throughput sequencing flow cell with four lanes (Illumina China, Shanghai, China).

Each library sequence was distinguished by the unique index. For each NIPS sample, approximately 5 million 36-bp reads were generated, of which approximately 3.5 million were uniquely mapped to the hg19 reference genome using RUPA extreme speed information analysis method. Z-score was calculated upon repetitive sequences removal, effective readings calculation, and GC correction. The fetal aneuploidy status for all 24 chromosomes was determined based on Z-scores (normal range, −3 < Z < 3). Lack of result in a sample was attributed to insufficient (<4%) fraction of cffDNA, unusually high variation in cffDNA counts, or failure to pass the quality control measures. Based on these results, chromosomal aneuploidy was determined in the fetus.

### Invasive prenatal diagnosis

After the NIPS test, all participants were given a general test report showing the estimated fetal risk (positive or negative) of trisomies 13, 18, and 21; a suspected risk of SCA was reported to the clinician in the form of a supplementary report. In such cases, post-test clinical counselling was offered by qualified clinical geneticists. Following the results of NIPS testing, pregnant women agreed to invasive prenatal diagnosis by amniocentesis or cordcentesis. Upon standard metaphase conversion of cultured fetal cells, amniotic fluid cells were cultivated using BIOAMF^TM^-3 medium (Biological Industries, Kibbutz Beit-Haemek, Israel) and AMINOPAN Medium(PAN-biotech GmbH, Aidenbach, Germany). The umbilical cord blood cells were cultured using HyClone RPMI Medium Modified (HyClone, USA) supplemented with heparin, PHA, and serum. More than 20 metaphase cells of each specimen were analysed at a resolution of 320 G-bands.

### Statistical analysis

The version 19.0 of Statistical Product and Service Solutions (SPSS) software (SPSS Inc., Chicago, IL, USA) was used to analyse the data. Data are presented in the form of mean ± SD. Anova was used for comparison between different groups. *P* values < 0.05 were considered statistical significant.

## Data Availability

No datasets were generated or analysed during the current study.
